# A novel mutation in *CELSR1* is associated with hereditary lymphedema

**DOI:** 10.1186/s13221-016-0035-5

**Published:** 2016-02-05

**Authors:** M. L. Gonzalez-Garay, M. B. Aldrich, J. C. Rasmussen, R. Guilliod, P. E. Lapinski, P. D. King, E. M. Sevick-Muraca

**Affiliations:** Center for Molecular Imaging, The Brown Foundation Institute of Molecular Medicine, The University of Texas Health Science Center, 1825 Pressler Street, SRB330A, Houston, TX 77030 USA; Memorial Herman Hospital and The University of Texas Health Science Center, Houston, TX 77030 USA; Department of Microbiology and Immunology, University of Michigan Medical School, Ann Arbor, MI 48109 USA

**Keywords:** Primary lymphedema, Whole exome sequencing, Near-infrared fluorescence imaging CELSR1, Planar polarity

## Abstract

**Background:**

Biological evidence reported in the literature supports the role of CELSR1 as being essential for valvular function in murine lymphatics. Yet thus far, there have been no variants in *CELSR1* associated with lymphatic dysfunction in humans.

**Case Presentation:**

In this report, a rare early inactivating mutation in *CELSR1* is found to be causal for non-syndromic, lower extremity lymphedema in a family across three generations. Near-infrared fluorescence lymphatic imaging shows that instead of being propelled within the lumen of well-defined lymphatic vessels, lymph moved in regions of both legs in an unusual fashion and within sheet-like structures.

**Conclusion:**

*CELSRI* may be responsible for primary, non-syndromic lymphedema in humans.

**Electronic supplementary material:**

The online version of this article (doi:10.1186/s13221-016-0035-5) contains supplementary material, which is available to authorized users.

## Background

Lymphedema (LE) is a condition caused by a defective lymphatic system where an excess of fluid accumulates and generates chronic swelling of tissues. Primary LE is a rare genetic disorder that can develop at birth (congenital), at puberty (praecox), or after 35 years of age (tarda) [1]. *FLT4* was the first pathogenic gene identified in primary LE [2], but to date, there is only a handful of genes (*FOXC2, CCBE1, GCJ2, SOX18, GATA2, PTPN14)* associated with hereditary LE, with the majority associated with patients in which LE is part of a genetic syndrome [3]. Mendola et al. recently argued that the genetic cause(s) of the majority of families harboring hereditary LE has not yet been identified. In their group of 78 index patients with inherited LE who were screened for mutations in the top seven LE genes, they found explanations for only 36 % of the cases [4].

In this report, we present the unusual lymphatic phenotype obtained by near-infrared fluorescence lymphatic imaging (NIRFLI) of a woman diagnosed non-syndromic, lower extremity LE and compare it with that of her asymptomatic mother. From clinical diagnoses and whole exome sequencing (WES) of family members across three generations, we found no gene variants known to be responsible for LE, but instead found a rare, inactivating mutation in *CELSR1* that could be causative for the inherited condition and the lymphatic phenotype imaged in the index case.

## Case Presentation

### Family case report

A 62 yo female previously diagnosed with primary, lower extremity LE (bilateral, Stage II by International Society of Lymphology staging system) and her 84 yo asymptomatic mother enrolled in an ongoing, institutional review board (IRB) and FDA-approved (IND# 102,827) clinical study of lymphatic disorders (Clinical Trials No. NCT00833599: “Imaging lymphatic function in normal subjects and in persons with lymphatic disorders” www.clinicaltrials.gov). The index case self-reported onset of lower extremity swelling at age 10, with diagnosis of LE at age 39 at which time she began compression treatment. In addition, she was also previously diagnosed with type 2 diabetes, high cholesterol, and high blood pressure. Following consent, a clinical examination was first performed followed by near-infrared fluorescence lymphatic imaging (NIRFLI) [5] to assess lymphatic anatomy and function in the legs. Blood was collected for WES of DNA.

Upon questioning, we further found a family history of lower extremity LE and traveled to the family’s hometown to consent, conduct standard clinical examinations (including measurements of leg volumes and assessment of Stemmer’s sign), and to collect blood from family members spanning three generations. We did not perform NIRFLI on these family members. Other than stage I and II bilateral lower extremity, non-syndromic LE, we found no remarkable evidence of disease among the family members.

### Imaging studies

**Fig. 1:**
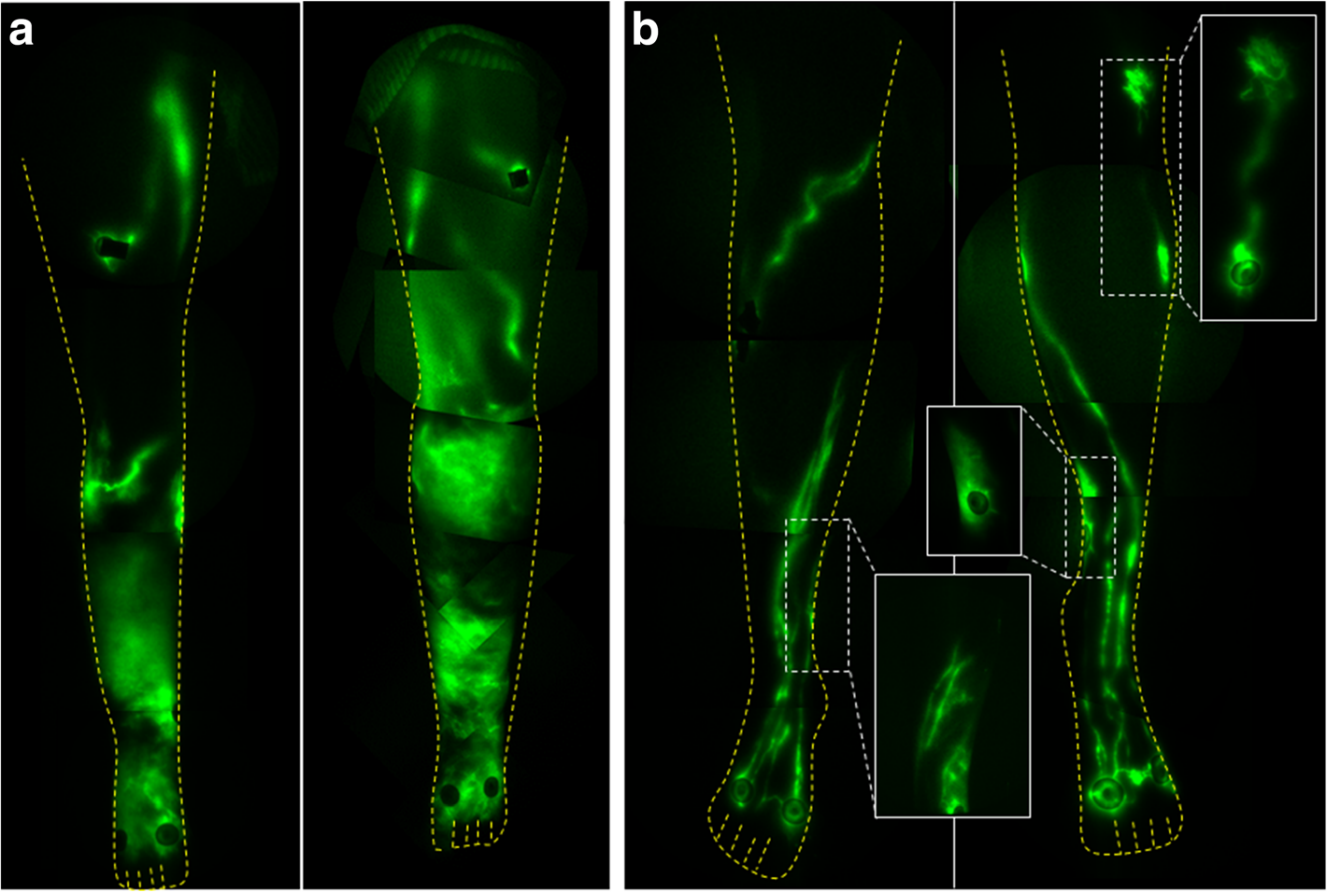
Montages of NIRFLI of the lower extremities of (**a**) the proband and (**b**) her mother. While the mother had well-defined lymphatics, she also had areas with dermal backflow as shown in the inset images of (**b**)

For lymphatic imaging of the daughter and mother, doses of 25 μg of indocyanine green (ICG) in 0.1 cc saline were administered bilaterally in the dorsum of the feet, medial ankles, the medial and lateral calves, and the thighs for a total dose of 300 μg. Images were acquired with exposure times of 200 ms and sequential images were compiled to assess for the presence of active lymphatic propulsion/movement of ICG-laden lymph from the injection sites proximally towards the inguinal lymph node basins. As shown by NIRFLI in Fig. 1b, the affected daughter had extensive areas of dermal backflow in her lower legs, as commonly observed in LE subjects, but possessed a unique, uncommon flow pattern of lymph movement that appeared in “sheets” rather than within distinct vessel lumens (Additional file 1: Video 1). While the proband’s mother primarily had well-defined and linear lymphatics (Fig. 1b) as typically seen in healthy subjects [6, 7], she nonetheless presented with smaller areas of dermal backflow and tortuous lymphatics as shown in the insets of Fig. 1b. Active lymphatic propulsion of ICG-laden lymph was observed in the well-defined lymphatic vessels in the mother (Additional file 2: Video 2), in contrast to the limited lymphatic movement observed in the daughter.

### Family evaluation

**Fig. 2:**
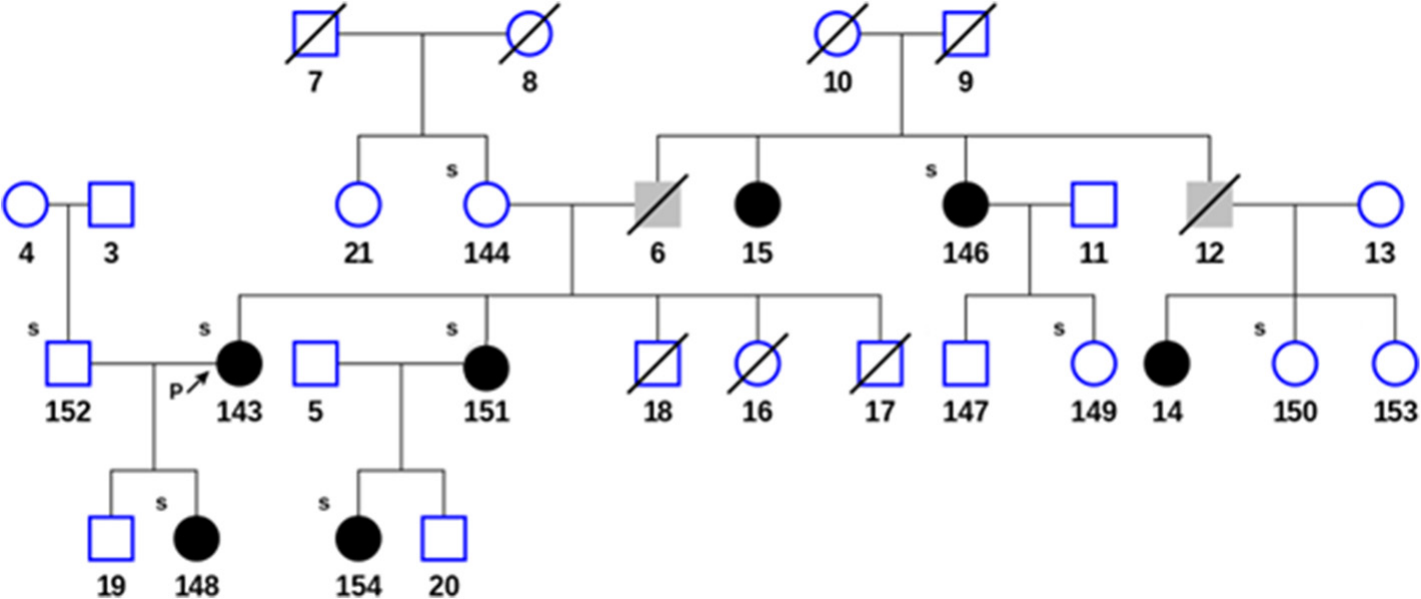
Pedigree of the family showing members diagnosed with lymphedema (filled black), unknown phenotype (filled gray) and unaffected (open) subjects. Arrow indicates the proband. The small ‘s’ at the left top corner of the subjects indicates when the subject’s DNA was sequenced. A diagonal line indicates that the patient is deceased

In addition to the proband and her mother, DNA was collected from 9 family members (subjects 146-154) including 5 members diagnosed with LE upon clinical examination. The pedigree chart in Fig. 2 shows that the diagnosed disorder segregates in an autosomal dominant manner. The affected members exhibited grade II bilateral leg lymphedema, with the exception of subject 154, with grade I bilateral leg lymphedema. DNA was extracted from blood samples using Paxgene Blood DNA Kit (PreAnalytix, Switzerland) according to the vendor's instructions. 2 μg of genomic DNA was submitted to Axeq Technologies for human exome capture sequencing using SureSelect Human All Exon V5 and V5 + UTRs (Agilent Technology, Santa Clara, CA).

### Genetic sequencing analyses

Genetic sequencing analysis was conducted as follows. Each pair of fastq files was aligned to human genome (hg19) using Novoalign (Novocraft Technologies; www.novocraft.com), keeping parameters at the default settings, as recommended by Novocraft Technologies. SAMtools (http://samtools.sourceforge.net) was used to sort the SAM files, create BAM files, and generate their index files. Picard (SourceForge; http://broadinstitute.github.io/picard) was used to remove all of the PCR duplicates from the BAM files. For local realignments, base quality recalibration, and variant calling, we used Genome Analysis Toolkit (GATK) Version 3.1-1 [8]. Finally, for variant annotation, we used SnpEff (http://snpeff.sourceforge.net/) [9], variant tools and ANNOVAR [10] using multiple databases from UCSC Genome bioinformatics. Functional effects for each non-synonymous coding variant was evaluated using three different functional prediction algorithms: (1) Polyphen 2.0 Prediction of functional effects of human nsSNPs (http://genetics.bwh.harvard.edu/pph2) [11], (2) SIFT [12] and (3) (www.mutationtaster.org) [13] using the dbNSFP database [14]. Filtration of common polymorphisms was accomplished using frequencies from the NHLBI Exome sequencing project (ESP) (http://evs.gs.washington.edu/EVS) [15] and the 1,000 Genomes Project (http://www.1000genomes.org/data) [16].

**Fig. 3:**
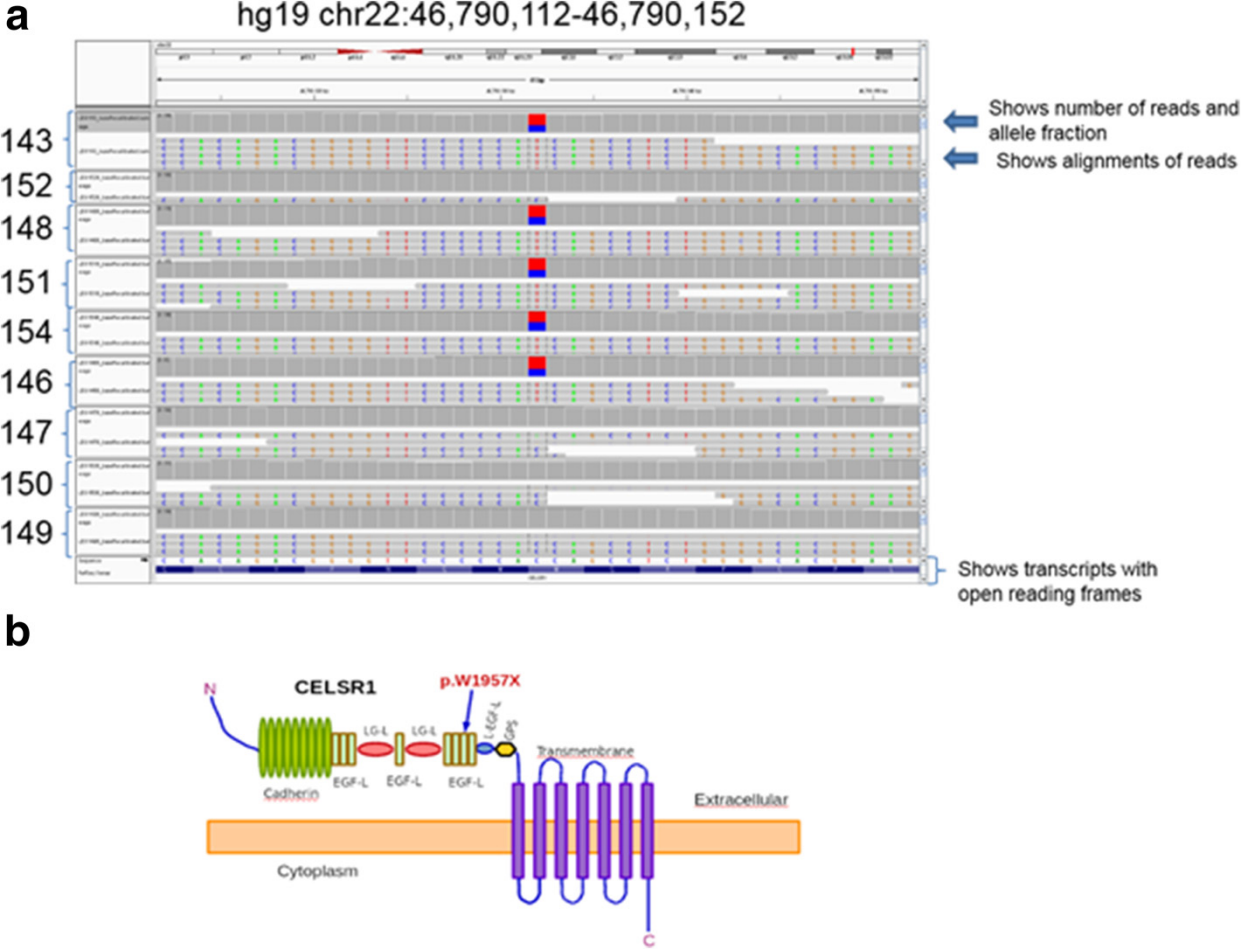
**a** Image from the Integrative genomics viewer, showing a 40 bp genomic region containing the variant. Each one of the samples is loaded into the panel. Each panel shows two windows, the top window displays the number of reads, and the allele fraction and the lower window show the alignments of the experimental reads. The bottom panel displays the transcripts with the open reading frames. **b** A cartoon representing the predicted protein structure of CELSR1 (uniprot id Q9NYQ6). An arrow represents the location of the mutation. The cartoon was inspired by the publication of Lei, et al.*,* 2014 [30]

WES results showed that the proband did not have variants in any of the known genes previously associated with lymphatic dysfunction. To maximize the power of the segregation analysis, we selected family members diagnosed with LE from each generation for a total of 5 affected cases. Since we did not have controls of the same ethnicity, we selected 4 unaffected family members to reduce the background and increase the statistical power of the analysis. The raw results from the sequencing passed our standard quality control, and the variant files (vcf) reported a consistent number of variants. After selecting non-synonymous coding variants with low frequency (MAF < 0.1), we performed co-segregation analysis using a dominant model of inheritance. Our analysis generated six potential candidate variants, five of them were eliminated after a quick manual inspection with the Integrative genomics viewer [17]. We identified a nonsense coding variant that generated a prematurely terminated protein CELSR1 (p.W1957X, Hg19chr22:g.46,790,132C > T; NC_000022.10:g.467901432C > T; NM_014246.1:c.5871G > A). The variant was private to the family and has not been reported in any of the following databases: 1000 Genomes [16], ESP6500 [15],UK10K, TCGA germline [18], Scripps Wellderly [19], ExAC [20], dbNSFP [14], ClinVar [21], OMIM [22], COSMIC [23], and Reference Variant Store (RVS), [24]. The variant sequence was of high quality with an average of 108.54 ± 20.93 reads per sample, mapping quality > 70, and Phred quality scores > 35. Furthermore, the variant was confirmed in each sample by Sanger sequencing in the forward direction using primer TGAGGTTGGGAGCCGGTAGAGG and the reverse direction using primer GTACCGACAGGGTATGTGAAGGCG (see Additional file 3: Figure S1). The segregation results were perfect, the variant was present in all the affected and was not present in any of the unaffected. We genotyped two additional unaffected members of the family, and they also lacked the variant. We searched using blat a 90 bp region containing the mutation against the human reference hg19 and found that the region was 100 % unique in the human genome. We also found that the same region was totally free of any repeats and evolutionarily conserved in vertebrates according to the comparative genomics track from the UCSC genome browser [25]. In summary, the ultra-rare damaging variant was present in five affected members and not present in six unaffected family members (Fig. 3a). From DNA of the family members sampled, there appears to be 100 % penetrance with an autosomal dominant pattern of inheritance.

## Discussion

CELSR1 is a member of the cadherin superfamily, conserved during evolution with orthologs in invertebrate and vertebrates [26]. In addition, CELSR1 is an atypical cadherin involved in planar cell polarity. This protein has nine cadherin repeats, eight EGF-like domains, and seven transmembrane segments (Uniprot_id Q9NYQ6). CELSR1 is located at the plasma membrane with the cadherin domains acting as homophilic binding regions and the EGF-like domains involved in cell adhesion and receptor-ligand interactions. We postulate that when the mutated allele is expressed, the protein lacks the entire cadherin domain, the five EGF domains, and the LamG domains (Fig. 3b).

*CELRS1* has an ortholog in Drosophila, the flamingo (Fmi). Fmi is localized at cell–cell boundaries in the wing. In the absence of Fmi, planar polarity was distorted [27]. In humans and rodents, there are three genes *CELSR1–3* and Celsr1–3. Celsr1 and Celsr2 expression is observed during gastrulation and within the developing nervous system. Celsr3 transcripts are found only at sites of active neurogenesis [28] and mutations in *CELSR1* are associated with neural tube defects in humans [29, 30].

Recent biological evidence reported in the literature supports the causal role of *CELSR1* for LE in the family pedigree. Tatin et al. recently showed that CELSR1 and VANGL2 play critical roles in the complex morphogenetic process of intraluminal valve formation in murine lymphatic vessels. In their work, they demonstrated that during valve leaflet formation, endothelial cells recruit CELSR1 and VANGL2 from filopodia to discrete membrane domains at cell-cell contacts. Furthermore, mouse mutants Crsh (Celsr1) and Looptail (Vangl2) show valve aplasia [31].

The proband displayed a common lymphatic phenotype consisting of extensive dermal backflow, tortuous vessels, as well as unique “sheet-like flow” in both legs indicating a defect in the valvular mechanism of lymph propulsion in collecting vessels. While we have not conducted functional studies of the *CELSR1* mutation identified in this family, the phenotype is consistent with the phenotype described in the mouse mutant Crsh [31].

In this family, the phenotype was not apparent in any living males, but obviously was passed to daughters through male parents, both now deceased and not available for clinical diagnosis or DNA collection. Three living males in the third and fourth generations were not reported to have lymphedema, but unfortunately not available for clinical diagnosis and genotyping. As a result, we are unable to determine whether penetrance is limited to females.

## Conclusion

This report provides the first evidence that defective planar cell polarity signaling pathway may participate as the cause of primary, non-syndromic lymphedema in humans.

### Consent

Written informed consent for images contained in this report was obtained from the patient's parents in accordance with the guidelines of the University of Texas Health Science Center Institutional Review Board.

## Additional files

Additional file 1: Video 1. The abnormal NIRFLI movie of lymphatic function in the left foot of the proband showing unusual lymph flow from the dorsal foot injections. (AVI 4088 kb) Additional file 2: Video 2. The normal NIRFLI movie of lymphatic function in the left foot of the asymptomatic mother showing typical lymph flow from the dorsal foot injections within collecting lymphatic vessels. (AVI 5480 kb) Additional file 3: Figure S1. A composite picture of two electropherograms comparing the Sanger sequencing results from two members of a family with lymphedema. The top panel represents the WT sequence of an unaffected family member (#152). The low panel represents the heterozygous mutation in the affected index patient (#143). The validation was performed as described previously [32] using the following primers: forward TGAGGTTGGGAGCCGGTAGAGG and reverse GTACCGACAGGGTATGTGAAGGCG. (TIF 201 kb)

## Abbreviations

ICG: indocyanine green
NIRFLI: near-infrared fluorescence lymphatic imaging
LE: lymphedema
