# Supplementary figures and images for: A novel mutation in CELSR1 is associated with hereditary lymphedema
Source: Vasc Cell. 2016 Feb 5;8:1. doi: 10.1186/s13221-016-0035-5 (PMC4743364; doi:10.1186/s13221-016-0035-5)

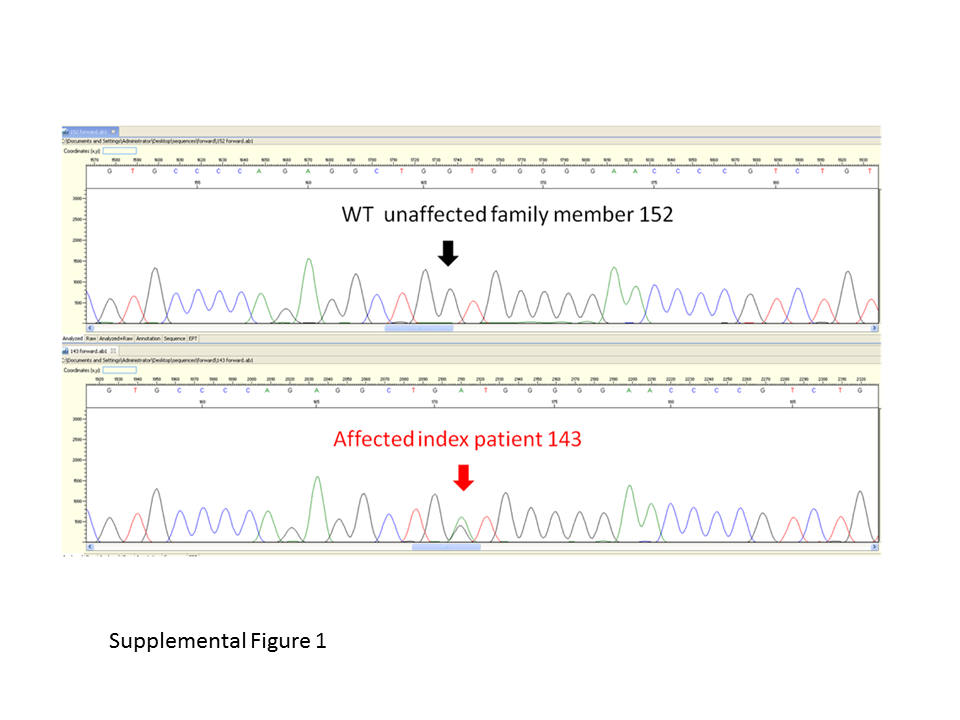

Supplement: Additional file 3: Figure S1. — A composite picture of two electropherograms comparing the Sanger sequencing results from two members of a family with lymphedema. The top panel represents the WT sequence of an unaffected family member (#152). The low panel represents the heterozygous mutation in the affected index patient (#143). The validation was performed as described previously [32] using the following primers: forward TGAGGTTGGGAGCCGGTAGAGG and reverse GTACCGACAGGGTATGTGAAGGCG. (TIF 201 kb) [file 13221_2016_35_MOESM3_ESM.tif]
